# Left atrioventricular coupling in isolated pediatric mitral valve prolapse with preserved ejection fraction and moderate regurgitation

**DOI:** 10.1038/s41598-025-33951-2

**Published:** 2026-01-20

**Authors:** Farah Ahmed Shokeir, Aya Mohammed Abdel Aziz, Rasha Karam, Ahmed Ibrahim Tawfik, Mahmoud Abdelbadie Salem, Aya Elboghdady

**Affiliations:** 1 Department of Radiology, Faculty of Medicine, Mansoura University, P.O. Box: 35516, El Gomhoria Street, Dakhlia governorate, Mansoura, Egypt; 2 Department of Cardiology, Mansoura University Faculty of Medicine, El Gomhoria Street, Dakhlia governorate, P.O. Box: 35516, Mansoura, Egypt

**Keywords:** Cardiac MRI, Feature tracking, Atrioventricular coupling, Left atrioventricular coupling index (LACI), Cardiology, Diseases

## Abstract

This study aimed to study the coupling between the left ventricle and left atrium in pediatric patients with mitral valve prolapse (MVP) by utilizing strain parameters and the left atrioventricular coupling index (LACI). A retrospective analysis was conducted over six years to identify patients with MVP and moderate mitral regurgitation who had undergone cardiac MRI. The study included 20 patients and 20 healthy controls. Functional and strain assessments of the left ventricle (LV), right ventricle (RV), and left atrium (LA) were performed and compared to those of healthy controls. LACI was determined by calculating the ratio of the LA end-diastolic volume indexed to the left ventricular end-diastolic volume indexed, and it was correlated with conventional CMR and strain parameters. Native T1 mapping was used as a non-contrast CMR technique to assess diffuse myocardial fibrosis in all patients. LACI revealed a negative correlation with left ventricular end-diastolic volume indexed to body surface area (LVEDVI) and total left atrial ejection fraction (LAEF) (*r* = -0.39, -0.65, *P* < 0.05, respectively). In addition, LACI showed a correlation with strain parameters, specifically with LA longitudinal peak positive strain rate and left ventricular global circumferential strain (LV GCS) (*r* = 0.35, -0.49, *P* < 0.05, respectively). The results from the multivariate regression analysis indicated that the left atrial longitudinal reservoir strain (ξs) was independently associated with left ventricular ejection fraction [β = 0.529; 95% confidence intervals (CI) = 0.192 to 0.866] and LV GRS [β = 0.229; 95% CI = 0.128 to 0.330]. There were statistically significant differences in LACI and LA ξs between patients who experienced clinical events such as arrhythmia and those who did not, with p-values of < 0.05. Strain parameters showed better results in evaluating LA and LV coupling in our patient group. Elevated native T1 values correlated with LACI, impaired LV, and LA strain parameters.

## Introduction

Mitral valve prolapse (MVP) is a prevalent valvular disorder that affects around 2–3% of the population. Although often deemed benign, growing evidence links MVP with severe complications like mitral regurgitation (MR), arrhythmias, myocardial fibrosis, and, in rare instances, sudden cardiac death^1^.

The histological structure of MVP is marked by myxomatous degeneration in the mitral leaflets, manifesting as leaflet billowing, malcoaptation, and stretching or rupture of chordae^2^. Recent insights indicate that the pathological changes associated with MVP extend beyond the valve itself and often include myocardial fibrosis, which is linked to arrhythmias and sudden cardiac death^3^.

Supporting evidence from various studies shows increased fibrosis in patients experiencing ventricular arrhythmias, and cardiac magnetic resonance imaging reveals that localized LV fibrosis is more common in MVP patients than those without MVP who have mitral regurgitation^4^.

Regardless of mitral regurgitation (MR) severity, evidence suggests intrinsic myocardial abnormalities in patients with MVP^5,6^.

Until recently, imaging of MVP was primarily done through echocardiography. However, in the past few years, it has become clear that cardiovascular magnetic resonance (CMR) has distinct advantages in detecting and measuring mitral valve abnormalities, as well as assessing myocardial tissue characteristics. Recent studies suggest that CMR is not just a substitute for echocardiography in patients with poor acoustic windows, but rather a supplementary imaging technique that is crucial for accurately measuring mitral valve abnormalities, the severity of mitral regurgitation, ventricular changes, and myocardial tissue alterations^7^.

Various structural and functional left ventricular parameters, such as left ventricular ejection fraction (LVEF), myocardial strain, the left ventricular mass-to-volume ratio, and the left ventricular global function index, have shown promise in predicting cardiovascular disease risks. Even with preserved LV systolic function, dysfunction of the left atrium (LA) can negatively impact overall heart performance, and a lack of coordination between the functionality of the two chambers may also contribute to cardiac dysfunction and clinical heart disease^8^.

The left atrioventricular coupling index (LACI) is a newly introduced metric that assesses the mechanical coordination between the left atrium and left ventricle, which is essential for optimal cardiac performance, expressed as the ratio of the minimum LA volume to the LV end-diastolic volume. In contrast to isolated strain metrics that necessitate complicated post-processing, LACI can be obtained from standard volumetric measurements and may act as a practical and comprehensive indicator of atrioventricular function^9^.

In MVP, the intrinsic myocardial abnormalities may lead to subclinical dysfunction of both chambers. Recent research has shown its effectiveness in risk assessment, especially among patients with mitral valve disorders and heart failure, where a raised LACI is linked to poor prognoses^9^.

Cardiac magnetic resonance (CMR) imaging, combined with strain analysis, offers a sensitive tool for evaluating early myocardial changes. Also, Native T1 mapping is a non-invasive CMR technique increasingly used for myocardial tissue characterization. It can detect diffuse myocardial fibrosis without the need for contrast, making it especially suitable in pediatric settings or where gadolinium use is limited. Prior studies have shown elevated native T1 values in cardiomyopathies and valvular diseases, reflecting early myocardial changes. In MVP, such alterations may contribute to unveiling subclinical dysfunction and arrhythmic risk^10,11^.

Most existing studies on MVP focus on adult populations, with limited data addressing myocardial involvement and atrioventricular interaction in pediatric patients.

This study aims to assess the left atrioventricular coupling in pediatric patients with isolated MVP using CMR-based strain and volumetric indices, including LACI. We also investigate how these parameters correlate with clinical events and myocardial functional status. Our findings offer new insights into early structural and functional alterations in pediatric MVP and their potential prognostic implications.

## Methods

### Study population

A retrospective analysis of the database was performed for all pediatric patients who underwent cardiac MRI studies from 2019 to 2024 and were identified as having isolated mitral valve prolapse along with a moderate level of mitral regurgitation. This approach ensured the homogeneity of our study group and eliminated the known effects of varying degrees of mitral regurgitation on our strain and functional results. Ultimately, out of 500 CMR studies, a total of 20 pediatric cases of isolated MVP diagnosed by Echocardiography (ECHO) with normal left ventricular ejection fraction and a moderate degree of MR^12,13^ were included in this study. Cardiac magnetic resonance (CMR) was also conducted on 20 healthy participants who had similar demographic characteristics to those in our study group. Exclusion criteria for the MVP group included patients with known structural heart diseases other than MVP, moderate-to-severe mitral regurgitation beyond the study’s defined range, MVP patients with impaired LV EF, history of cardiac surgery or intervention, known cardiomyopathies, arrhythmia-related myocardial remodeling such as tachycardiomyopathy, or systemic illnesses (e.g., autoimmune, metabolic, or neuromuscular disorders), heart failure with preserved ejection fraction, or inadequate CMR image quality or incomplete clinical data. Exclusion criteria for controls included any known cardiovascular disease (congenital or acquired), abnormal electrocardiogram, or echocardiographic findings, history of systemic illness (e.g., diabetes, hypertension, chronic kidney disease), family history of genetic cardiomyopathies or sudden cardiac death, or any medication use that could affect cardiovascular function. All control participants were asymptomatic, had no history of cardiac complaints, and underwent a thorough review of medical records to ensure they were free from significant clinical conditions. Clinical endpoints were established as the incidence of arrhythmias, hospitalizations associated with cardiac events, or mortality during the follow-up period. Arrhythmias were identified and categorized based on documented clinical records and electrocardiographic findings, including types such as ventricular ectopy, supraventricular arrhythmias, and other clinically significant rhythm disorders. Hospitalizations were recorded exclusively if they were related to cardiovascular events. No fatalities were reported during the study period. Patients underwent regular clinical evaluations and ECG monitoring at predefined intervals, with a minimum follow-up duration of 18 months. This follow-up protocol facilitated consistent assessment and documentation of clinical events. This study was approved by the Institutional Review Board (IRB) of Mansoura University Faculty of Medicine (IRB ID: R.23.09.2327). Informed consent was waived due to the retrospective nature of the study. All procedures adhered to the guidelines set forth by the World Medical Association Declaration of Helsinki.

### Clinical data

Demographic and clinical information were collected from electronic medical records retrieved from the Mansoura University Children’s Hospital, Cardiology unit. The included patient outcomes tracked over a minimum period of 18 months, including the occurrence of arrhythmia, hospital admissions, or death, as well as the necessity for implantable cardioverter defibrillator (ICD) placement.

### Cardiac MRI protocol

All participants underwent CMR using a 1.5-T magnet (Inginea, Philips release 4, Netherlands). The slices were obtained in axial, short-axis, two, three, and four-chamber planes during different phases of the cardiac cycle using a free-breathing retrospective ECG-gated steady-state free precession (SSFP) sequence. The following parameters were used: time of repetition (TR) = 3.2–3.65 msec, time to echo (TE) = 1.6–1.83 msec, field of view (FOV) = 270 mm2, slice thickness = 5 mm, 30 cardiac phases, no slice gap.

### CMR functional and strain analysis

The functional and strain analysis of both LA and LV was done using commercial software (Cvi42-v5.16; Circle Cardiovascular Imaging, Inc., Calgary, AB, Canada).

All CMR functional and strain analyses were conducted by an experienced cardiac radiologist with 12 years of expertise (FS and AE). To account for interobserver variability, a second radiologist repeated the measurements for a selection of randomly selected subset of 10 cases. Intraclass correlation coefficients (ICCs) were calculated to evaluate interobserver and intraobserver variability.

#### Biventricular analysis

The quantification of biventricular volumes, EF, and LV end-diastolic wall mass was assessed from bSSFP-cine images by automatically tracing the endocardial contours of both ventricles and the epicardial contour of LV in the short axis view in end-diastole and end-systole, followed by manual correction if required. All measures were indexed to body surface area.

**Fig. 1:**
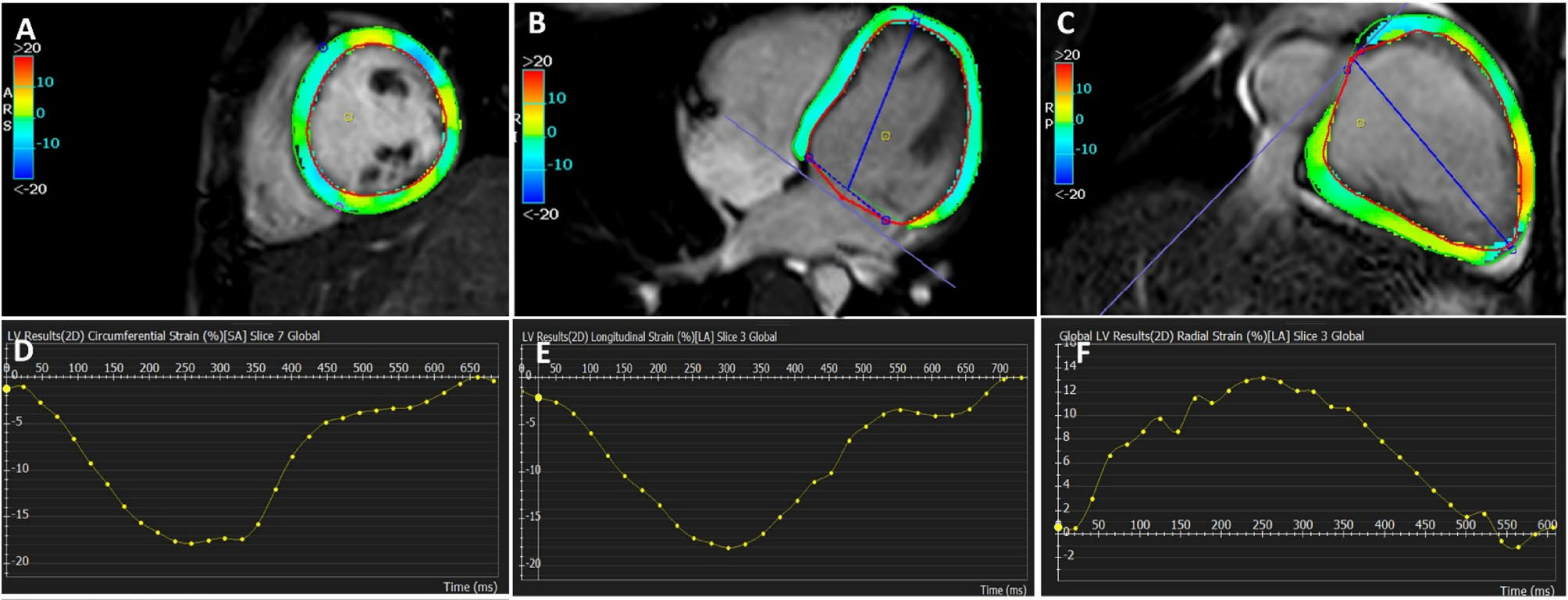
Analysis of CMR left ventricular strain showing (**A**) LV GCS in short axis view, (**B**) LV GLS color map in four-chamber view, (**C**) LV GRS in two-chamber view, (**D**) LV GCS curve, (**E**) LV GLS curve, (**F**) LV GRS curve. *LV *left ventricle,* GCS *global circumferential strain,* GLS *global longitudinal strain,* GRS *global radial strain*.*

For LV strain analysis, endocardial and epicardial contours of the LV were automatically traced and manually corrected if needed at the end-diastolic phase in short-axis planes and long-axis planes (four, two, and three-chamber planes). Global longitudinal strain (GLS) and global radial strain (GRS) analyses were performed using long-axis images, while global circumferential strain (GCS) analyses were performed using short-axis images. Strain values, strain curves, polar maps, and color-coded images were obtained (Fig. 1).

#### LA analysis

**Fig. 2:**
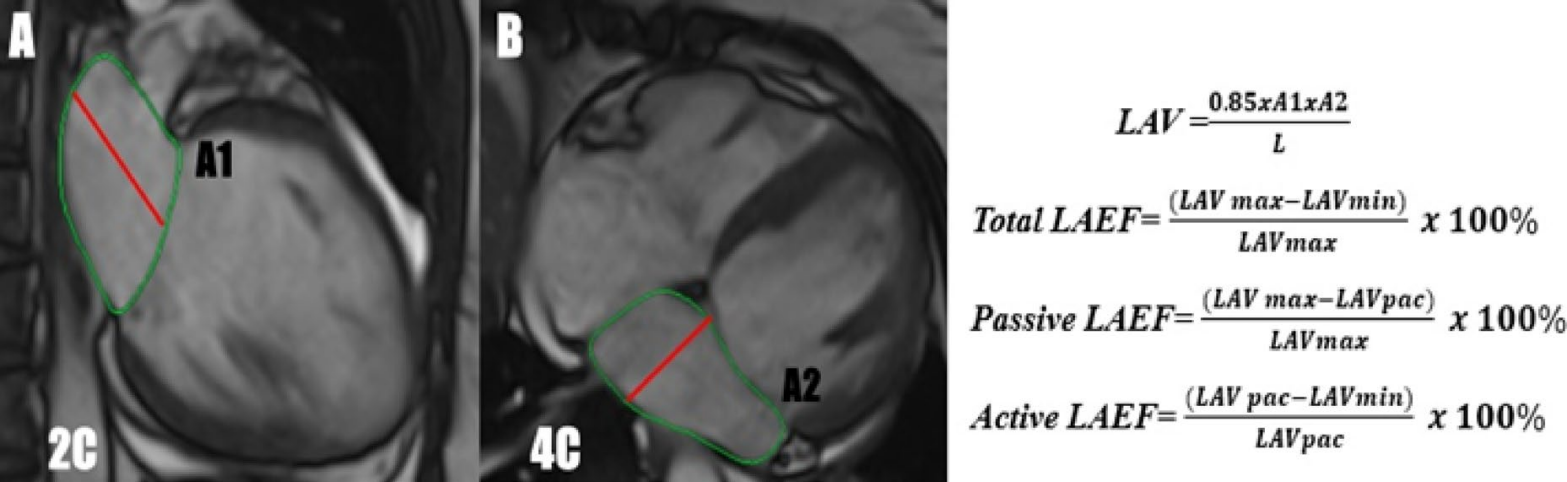
Calculation of left atrial volume and function. The left atrial volume was calculated by the biplane method, where A1&A2 were areas measured in 2-chamber and 4-chamber views respectively. L was the linear measurement acquired parallel to the atrial septum or perpendicular to the mitral annulus. Images (**A** and **B**) demonstrate how the left atrial volume was measured at the end of left ventricular systole (LAV max). The left atrial ejection fraction was calculated according to the mentioned formulas. *2 C *2 chambers,* 4 C *4 chambers,* LAV max *left atrial volume maximum,* LAV min *left atrial volume minimum,* LAV pac *left atrial volume pre-atrial contraction,* LAEF *left atrial ejection fraction*.*

LA function was analyzed by tracking the endocardial contour of LA in two long-axis planes (four and two-chamber planes) during the whole cardiac cycle. The left atrial volume was calculated by the biplane method following the formula: LA Volume = 0.85XA1xA2/L, where A1&A2 were areas measured in 2 C and 4 C views, respectively. L was the linear measurement acquired parallel to the atrial septum or perpendicular to the mitral annulus. LA volume was calculated three times: at the end of left ventricular systole (LAV max), before left atrial systole (LAV pac), and at the end of left ventricular diastole (LAV min). LA function indices were calculated, including total left atrial emptying fraction (LA EF), passive LA EF, and active LA EF, as shown in (Fig. 2).

**Fig. 3:**
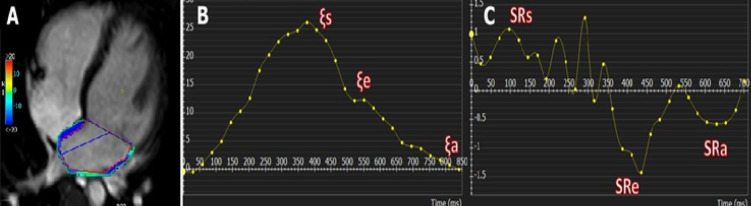
Analysis of CMR left atrial strain showing (**A**) LA longitudinal strain color map in four-chamber view, (**B**) LA longitudinal strain curve, and (**C**) LA longitudinal strain rate curve. *LA *left atrium*.*

Analyzing the LA strain was done by manually tracing the endocardial and epicardial contours of LA in the end-diastolic and end-systolic phases in the 4-chamber view, and then the CMR-FT software automatically tracked the border across the whole cardiac cycle. Multiple manual corrections were required to achieve good-quality tracking. The software automatically generated the curve of the global longitudinal and radial strains of LA. From the longitudinal strain curve, additional left atrial strain indices were derived: longitudinal reservoir strain (filling, ξs), which reflects the LA’s storage function during ventricular systole; passive strain (conduit, ξe), representing LA emptying during early ventricular diastole; and active strain (booster, ξa), which reflects LA contraction in late diastole. From the longitudinal strain rate curve, we derived the following indices were obtained to further assess the dynamic function of the left atrium^8^: Reservoir strain rate (SRs): The peak positive strain rate during ventricular systole, reflecting the rate at which the left atrium fills while the mitral valve is closed, Conduit strain rate (SRe): The early peak negative strain rate during early diastole, representing the rate of passive emptying of the LA into the LV and Booster strain rate (SRa): The late peak negative strain rate during atrial contraction (late diastole), reflecting the contractile (booster pump) function of the LA as shown in (Fig. 3).

#### Left atrioventricular coupling index

LA and LV volumes were measured in the same end-diastolic phase as previously described and indexed to the body surface area. LACI was defined as the ratio of the minimal left atrial volume indexed to the left ventricular end-diastolic volume indexed (LVEDVI) assessed through cardiac magnetic resonance imaging (CMR)^14^.

### T1 mapping analysis

Native T1 mapping was performed using a modified Look-Locker inversion recovery (MOLLI) sequence on all patients and controls to assess myocardial tissue characteristics. Post-contrast T1 mapping and extracellular volume (ECV) quantification were not available in this retrospective cohort. Native T1 values were measured in the mid-ventricular septum, avoiding areas with artifact or fibrosis. The correlation between native T1 values and left atrioventricular coupling index (LACI), as well as other functional parameters, was assessed to explore tissue-level alterations.

### Statistical analysis

Continuous variables were presented as the median with the upper and lower limits (75th and 25th percentiles). Student’s t-test or Mann-Whitney U test was used for comparing two groups, while one-way analysis of variance was utilized for comparing more than two groups. The categorical variables were represented using frequency percentages, and a Chi-square test was employed to compare the ratios of components among various groups. Spearman’s test was utilized to examine the correlation between LA strain, LV function indices, and LACI. Additionally, a multivariate analysis was conducted to assess the relationship between LA strain and LV indices. A ROC curve was performed to detect the cutoff value of LACI between MVP patients with clinical events, such as arrhythmia, and those without. Another cutoff point was determined using LA ξa to sort patients who suffered arrhythmia from those who didn’t. The cutoff point was based on the highest sensitivity and specificity, and for combined parameters, with the calculation of area under the curve (AUC), 95% CIs, sensitivity, and specificity. All statistical analyses were conducted using a two-tailed test, with a significance level of *P* < 0.05.

## Result

### Demographic characteristics of the study cohort

(Table 1) shows the demographic characteristics of patients versus control subjects. There was no statistically significant difference in age between MVP patients [11 (6.6–13) years] and controls [10.5 (5–14) years]. No significant preference was for any specific gender (P value = 0.736). As for weight, height, and BSA, there was no statistical significance between patients and controls as follows: Weight [31 (17.75–50) kg vs. 30 (21–47.25) kg], height [1.35 ± 2.6 m vs. 1.33 ± 2.7 m], and BSA [1.10 ± 0.40 m^2^ vs. 1.11 ± 0.36 m^2^].

**Table 1:** Analysis of demographic data in the study groups.

Variables		MVP group (*n*= 20)	Control group (*n*= 20)	*P* value
Age (years)		11 (6.625–13)	10.5 (5–14)	0.755
Sex	Males	6 (30%)	7 (35%)	0.736
	Females	14 (70%)	13 (65%)	
Weight (kg)		31 (17.75–50)	30 (21–47.25)	0.978
Height (m)		1.35 ± 2.6	1.33 ± 2.7	0.867
BSA (m^2^)		1.10 ± 0.40	1.11 ± 0.36	0.944

*NB* Quantitative data are expressed as mean ± SD (Independent samples t-test). Quantitative data are expressed as median (IQR) (Mann-Whitney U-test). Qualitative data are expressed as numbers (percent) (chi-square test). *BSA* body surface area.

### Comparison of ventricular and atrial CMR and strain indices between MVP patients and normal individuals

Patients with MVP exhibited larger LV EDVI values [91 (83–136) ml/m²] compared to the control group [73.5 (64.5–89.5) ml/m²]. Additionally, the left ventricular end-diastolic wall mass (LV EDWM) was significantly larger in MVP patients [65 (38–122) gm] versus the control group [30 (19–35.5) gm], as was the LV end-diastolic wall mass indexed to body surface area (LV EDWMI) [32.1 (17.6–36.2) gm/m²] compared to [67.2 (35.9–188) gm/m²]. Ventricular functional analysis in patients and the control group is discussed in detail in (Table 2).

**Table 2:** Ventricular functional analysis in study groups.

Variables	Control group (*n*= 20)	MVP group (*n*= 20)	*P* value
LV EDV (ml)	70 (52.5–133)	106 (67–187.25)	0.079
LV EDVI (ml/m^2^)	73.5 (64.5–89.5)	91 (83–136)	< 0.001*
LV ESV (ml)	24.5 (15.5–42)	41.5 (17.25–75.75)	0.159
LV ESVI (ml/m^2^)	22.2 (11–28.7)	19.8 (11–114)	0.088
LVEF (%)	66.50 ± 3.80	64.95 ± 13.09	0.021*
LV wall mass (gm)	30 (19–35.5)	65 (38–122)	0.012*
LV wall mass index (gm/m^2^)	32.1 (17.6–36.2)	67.2 (35.9–188)	0.001*
RV EDV (ml)	79 (54.75–132.25)	89 (60.5–129.75)	0.655
RV EDVI (ml/m^2^)	70 (29–98)	69.5 (50.9–107)	0.245
RV ESV (ml)	32 (20–46.75)	42.5 (24.5–61)	0.330
LV ESVI (ml/m^2^)	25.2 (9.9–55.8)	21 (9.4–70.8)	0.074
RVEF (%)	63.25 ± 6.62	58.65 ± 11.12	0.120
Native T1 Mapping (ms)	934 (924,958)	1062(920,1100)	0.006*

*NB* Quantitative data are expressed as mean ± SD (Independent samples t-test). Quantitative data are expressed as median (IQR) (Mann-Whitney U-test). *: Statistically significant, LV: left ventricle, RV: right ventricle, EDV: end-diastolic volume, EDVI: end-diastolic volume indexed, ESV: end-systolic volume, ESVI: end-systolic volume indexed, EF: ejection fraction.

The minimum left atrial volume (LAV min) was also greater in MVP patients [22.5 (15.25–41) ml] than in the control group [12.5 (11–14) ml]. Interobserver and intraobserver reproducibility of volumetric measurements, including left ventricular end-diastolic volume (LVEDV), left ventricular end-systolic volume (LVESV), and left atrial volumes, was assessed using intraclass correlation coefficients (ICCs). The ICC values for volumetric parameters demonstrated good to excellent reliability, ranging from 0.85 to 0.93 for interobserver agreement and 0.87 to 0.95 for intraobserver agreement. These findings confirm that the volumetric measurements used in this study are robust and consistent across different observers and repeated measurements. The detailed atrial functional parameters in the study groups are illustrated in (Table 3).

**Table 3:** Atrial functional parameters in study groups.

Variables	Control group (*n*= 20)	MVP group (*n*= 20)	*P* value
LAV max indexed (ml/m^2^)	32.5 (27.25–33.75)	44.5 (30.25–76)	0.127
LAV min indexed (ml/m^2^)	12.5 (11–14)	22.5 (15.25–41)	< 0.001*
Total LA EF (%)	60 (53–64.9.9)	49.6 (42.2–65)	0.174
Passive LA EF (%)	32.4 (22.5–48.3)	31.6 (22.7–35.6)	0.758
Active LA EF (%)	42 (31–45)	28.6 (16.9–48.3)	0.602
LACI (%)	42.3 9(25–55.4.4)	41 (32–51)	0.752

NB: Quantitative data are expressed as median (IQR) (Mann-Whitney U-test)*: Statistically significant. LAV max: left atrial volume maximum, LAV min: left atrial volume minimum LA: left atrium, EF: ejection fraction, PAC: pre-atrial contraction, LACI: left atrioventricular coupling index.

Regarding strain values, patients with MVP showed lower LA GCS [−15.4 (−19, −12.4) %] compared to the healthy control group [12.8 (−14.6, −11.07) %]. They also had lowered LA GRS [26.9 (23.2–36.8) %] compared to [48 (34–66.4) %] and lower LA reservoir strain (ξs) [14.4 (10.5–17.7) %] compared to [21.2 (14.4–22.4) %]. Conversely, LV GLS was less negative in MVP patients [–15.4% (–15.6 to − 14.2) %] compared to controls [–18.6% (–19.3 to − 16.1)], indicating reduced myocardial deformation. Similarly, LA GLS was less negative [−15 (−16.9, −13.1) %] compared to [−18 (−24, −15.6) %]. Additionally, LA SRe was lower in this group [−0.8 (−0.8, −0.4) %] compared to [−2.05 (−2.6, −1.4) %], and LA SRa was also lower [−1.1 (−1.2, −0.6) %] compared to [−1.4 (−2.07, −0.72) %]. The remaining indices of patients with MVP were similar to healthy individuals without MVP (all *P* > 0.05). To assess reproducibility, ICCs were calculated for both interobserver and intraobserver variability using a subset of 10 randomly selected cases. The ICCs for LA strain parameters ranged from 0.81 to 0.89, and for LV strain parameters from 0.83 to 0.91, indicating good to excellent reproducibility. The correlation of LV and LA strain parameters between patients and the control group is presented in (Table 4).

**Table 4:** Comparison of LV and LA CMR strain parameters between the study groups.

	Control group (*n*= 20)	MVP group (*n*= 20)	*P* value
LV strain parameters			
GLS (%)	−18.6(−19.3,−16.1)	−15.4(−15.6,−14.2)	0.001*
GCS (%)	−12.8(−14.6, −11.07)	−15.4(−19, −12.4)	< 0.001*
GRS (%)			< 0.001*
LA strain parameters			
GLS (%)	−18 (−24, −15.6)	−15 (−16.9, −13.1)	0.021*
GRS (%)	48 (34, 66.4)	26.9(23.2,36.8)	< 0.001*
LA ξs (reservoir) (%)	21.2)14.4,22.4)	14.4(10.5,17.7)	< 0.001*
LA ξe (conduit) (%)	15.7 (11.5,21.6)	11.2(2.6,13)	0.002*
LA ξa (booster) (%)	5.9(4,6.5)	4.1 (2.4,4.7)	0.015*
LA strain rate parameters			
LA SRs (reservoir) (s^−1^)	1.7(0.9,1.2)	0.9(0.6,1)	0.007*
LA SRe (conduit) (s^−1^)	−2.05(−2.6,−1.4)	−0.8(−0.8, −0.4)	0.001*
LA SRa (booster) (s^−1^)	−1.4(−2.07,−0.72)	−1.1(−1.2, −0.6)	0.042*

Quantitative data are expressed as mean ± SD (Independent samples t-test). Quantitative data are expressed as median (IQR) (Mann-Whitney U-test). *: Statistically significant, LV: left ventricle, GLS: Global longitudinal strain, GCS: Global circumferential strain, GRS: global radial strain, LA: Left atrium, LA ξs: Left atrial longitudinal reservoir strain, LA ξe: Left atrial passive strain, LA ξa: Left atrial active strain, LA SRs: Left atrial longitudinal peak positive strain rate, LA SRe: Left atrial peak early negative strain rate, LA Sra: Left atrial peak late negative strain rate.

### Left atrioventricular coupling in MVP patients (correlation between LA strain and LV function)

**Fig. 4:**
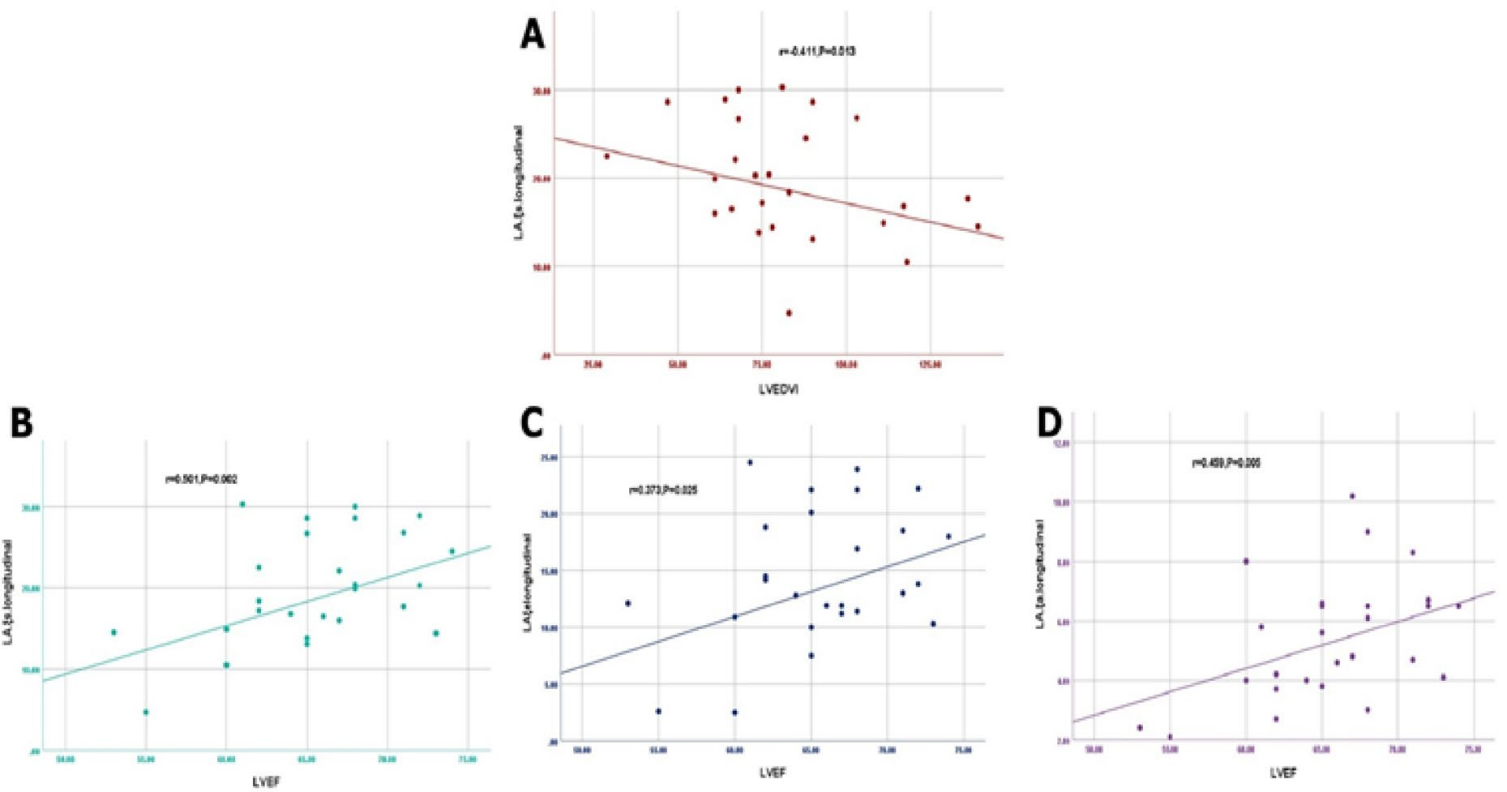
Scatter plots showing: (**A**) LA ξs negative correlation with LVEDVI, (**B**) LA ξs positively correlated with LVEF, (**C**) LA ξe positive correlation with LVEF, and (**D**) LA ξa positively correlated with LVEF. *LA ξs *left atrial longitudinal reservoir strain,* LVEDVI *left ventricular end-diastolic volume indexed to body surface area,* LVEF *left ventricular ejection fraction,* LA ξe *left atrial passive strain (conduit),* LA ξa *left atrial active strain (booster)*.*

Firstly, LA longitudinal reservoir strain (ξs) was negatively correlated with LVEDVI (*r*=−0.411, *P* < 0.05) and positively correlated with LVEF (*r* = 0.50, *P* < 0.05) in MVP patients. No correlation was found between longitudinal reservoir strain (ξs) and left ventricular end-systolic volume indexed to body surface area (LVESVI) and LV wall mass (*r* = 0.02, *r* = 0.12, respectively). Moreover, there was a positive correlation between LA passive strain (conduit, ξe) and LVEF (*r* = 0.37, *P* < 0.05). Additionally, the LA active strain (booster, ξa) was positively correlated with LVEF (*r* = 0.46, *P* < 0.05). No correlation was found between the passive strain (conduit, ξe) and LVESVI and LV wall mass (*r* = 0.23, *r* = 0.17, respectively) (Fig. 4).

### Correlation of left atrioventricular coupling index (LACI) with LA, LV functions, and strain parameters in MVP patients

**Fig. 5:**
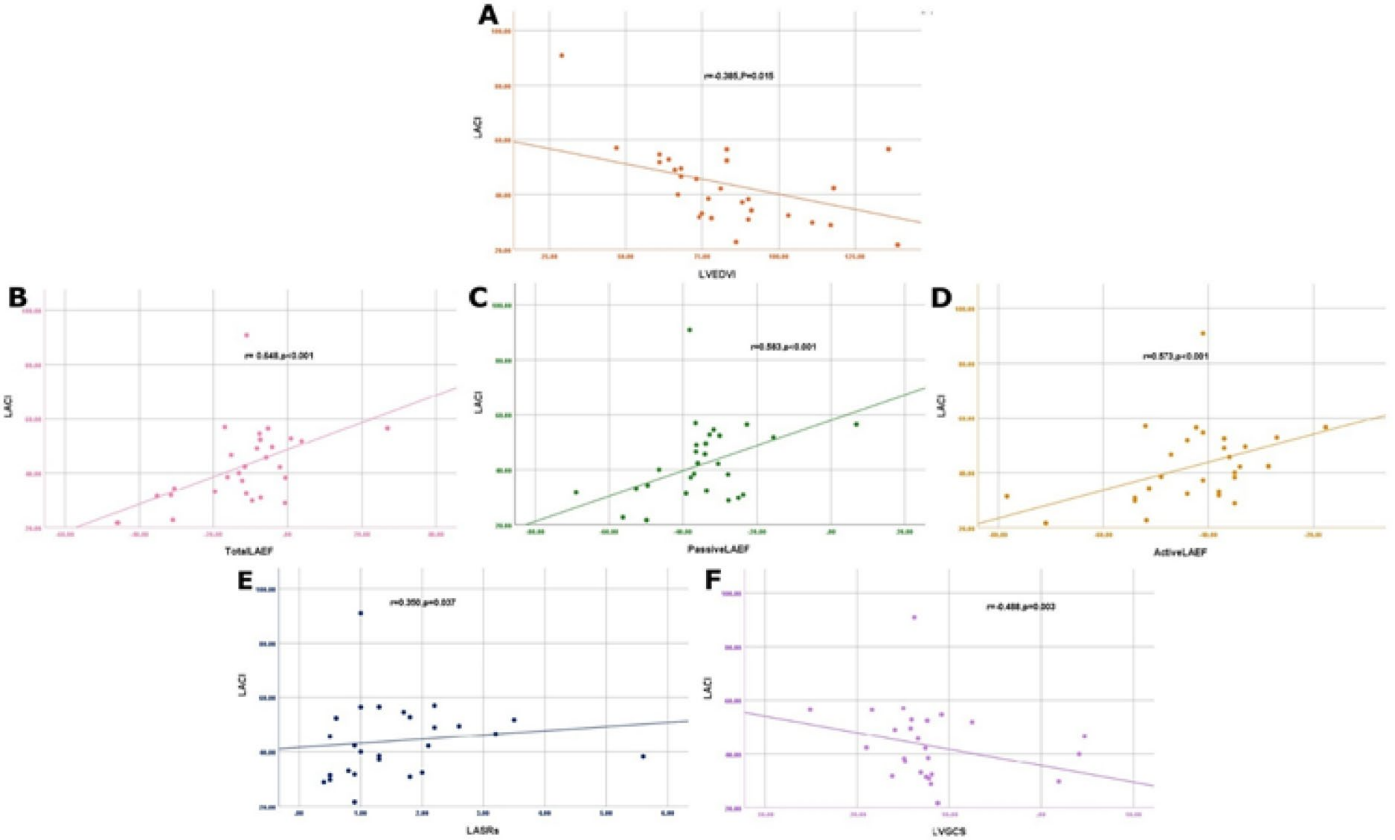
Scatter plots showing: (**A**) LACI negative correlation with LVEDVI, (**B**) LACI positive correlation with total LAEF, (**C**) LACI positive correlation with passive LAEF, (**D**) LACI positive correlation with active LAEF, (**E**) LACI positive correlation with LA SRs, and (**F**) LACI negative correlation with LV GCS. *LACI *left atrioventricular coupling index,* LAEF *left atrial ejection fraction,* LA SRs *left atrial strain rates,* LV GCS *left ventricular global circumferential strain*.*

Regarding correlations between LACI and the functions of LA and LV. LACI negatively correlated with LVEDVI (*r* = −0.39, *P* < 0.05). In contrast, LACI positively correlated with total LAEF (*r* = 0.65, *P* < 0.05), and passive and active LAEF (*r* = 0.58, *r* = 0.57, and *P* < 0.05, respectively). Additionally, LACI demonstrated a correlation with strain indices, specifically with LA SRs (*r* = 0.35, *P* < 0.05) and LV GCS (*r* =−0.49, *P* < 0.05). (Fig. 5).

### Independent influencers of LA strain parameters; multivariate analysis of left atrioventricular coupling indices; between LA strain and LV function in MVP patients

The multivariate regression analysis showed that the LA longitudinal reservoir strain (ξs) was independently correlated with the LVEF (β = 0.529; 95% confidence intervals (CI) = 0.192 to 0.866) and LV GRS (β = 0.229; 95% CI = 0.128 to 0.330). Furthermore, the LA passive strain (conduit phase, ξe) was independently correlated with LVEF (β = 0.370; 95% CI = 0.049, 0.692) and LVGRS (β = 0.198; 95% CI = 0.102 to 0.293). Finally, the LA active strain (booster phase, ξa) was also independently correlated with the LVEF (β = 0.158; 95% CI = 0.046 to 0.27) (Table 5).

**Table 5:** Multivariate analysis between LA strain and LV indices in patients with isolated MVP.

	LA ξs (reservoir) (%)			LA ξe (conduit) (%)			LA ξa (booster) (%)		
	Β	95% CI for β	*P*	β	95% CI for β	*P*	Β	95% CI for β	*P*
LVEDVI (ml/m2)	−0.054	−0.127, 0.020	0.148	−0.054	−0.124,0.016	0.126	0.001	−0.024,0.025	0.960
LVEF (%)	0.529	0.192, 0.866	< 0.05*	0.370	0.049,0.692	< 0.05*	0.158	0.046,0.27	< 0.05*
LV GCS (%)	−0.210	−0.381,1.006	0.203	−0.182	−0.494,0.129	0.241	−0.028	−0.154,0.099	0.661
LV GLS (%)	0.313	−0.539,0.119	0.365	0.357	−0.299,1.013	0.276	−0.044	−0.311,0.223	0.739
LV GRS (%)	0.229	0.128,0.330	< 0.05*	0.198	0.102,0.293	< 0.05*	0.031	−0.008,0.070	0.111

LA ξs Left atrial longitudinal reservoir strain, LA ξe Left atrial passive strain, LVEDVI left ventricle end-diastolic volume indexed, LVEF left ventricle ejection fraction, LV GCS left ventricle global circumferential strain, LV GLS left ventricle global longitudinal strain, LV GRS left ventricle global radial strain. Significant values are in bold.

### Clinical impact of left atrioventricular coupling index in MVP patients

**Fig. 6:**
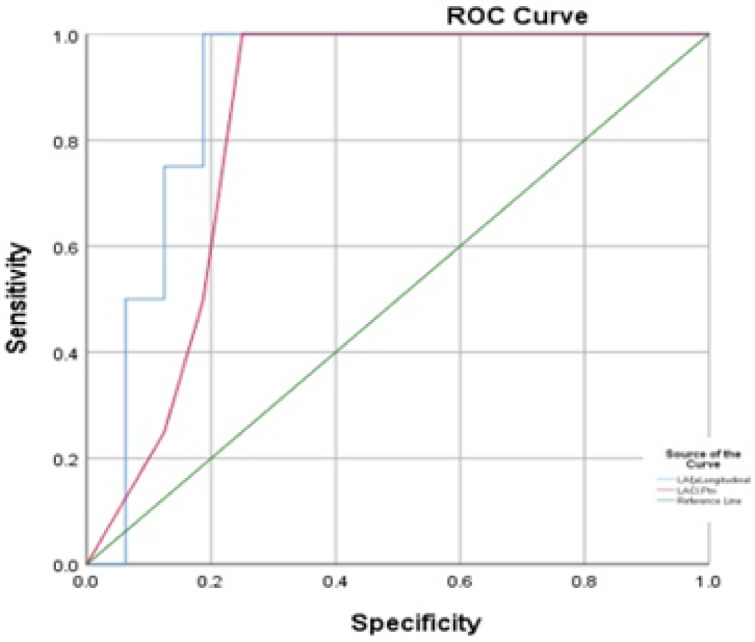
ROC analysis of LACI and of LA ξa in MVP patients who had clinical events as arrhythmia versus those who hadn’t (AUC 0.84, cutoff point = 38.3%), (AUC 0.89, cutoff point = 4.7), respectively. *LACI *left atrioventricular coupling index,* LA ξa *left atrial longitudinal reservoir strain*.*

The clinical outcomes, including arrhythmia occurrence, were followed over a median duration of a minimum of 18 months for the entire cohort. In the study cohort, a total of four patients reported experiencing arrhythmias, but these incidents were confined to benign forms of ventricular or supraventricular ectopy. Notably, there were no patients identified with sustained arrhythmias, such as atrial fibrillation or frequent premature ventricular contractions that could lead to cardiomyopathy. Among the four MVP patients who experienced arrhythmic events during follow-up, the arrhythmias observed included: two cases of isolated ventricular premature complexes, one case of supraventricular ectopy, and one case with both infrequent VPCs and short runs of nonsustained supraventricular tachycardia. None of the affected children exhibited symptoms warranting hospitalization or emergency care. All patients with arrhythmias were monitored conservatively. No anti-arrhythmic medications were initiated. Instead, regular follow-up with Holter monitoring and clinical assessment was maintained. Lifestyle guidance was provided, and none of the patients showed progression of arrhythmias during the follow-up period. Patients who suffered arrhythmia had a median LACI of 54.4%, whereas those who did not suffer any clinical events, including arrhythmias, had a median LACI of 34.2% and a P value of < 0.05. A ROC curve analysis yielded an AUC of 0.84 with a cut-off point = 38.3% showing sensitivity and specificity of 100% and 60% respectively. We conducted correlation analyses of other strain and volumetric parameters between the two groups, but there were no significant differences between them (Table 6). Additionally, the LA ξa differed significantly between the two groups with a P value of < 0.05. A receiver operating characteristic (ROC) curve analysis yielded an AUC of 0.89 with a cut-off point = 4.7, showing sensitivity of 100% and specificity of 60%. There were no records of hospital admissions or deaths in our study cohort (Fig. 6).

**Table 6:** Medians of patients exposed to a clinical event vs non-exposed patients.

Parameters	Exposed	Non-exposed	*P* value
LACI (%)	54.4 (52.4,56.56)	34.23 (21.67,56.56)	< 0.05*
LAV max indexed (ml/m2)	45.5(44,69.5)	31(30,50)	0.25
LAV min indexed (ml/m2)	23(22,34.5)	23(19,31)	0.55
LA ξs (reservoir) (%)	12(9.4,19.8)	14.5(9,18.4)	0.82
LA ξe (conduit) (%)	2.3(0.8,6.6)	4(2.3,11.7)	0.56
LA ξa (booster) (%)	5.35 (3.7,6.5)	6.15 (2.7,10.2)	**< 0.05**
LA SRs (reservoir) (s-1)	1(0.7,1.5)	1(0.6,1.9)	1.00
LA SRe (conduit) (s-1)	−1.4(−1.5, −0.9)	−0.8(−1.6, −0.5)	0.39
LA SRa (booster) (s-1)	−1.7(−2.1, −1.3)	−0.8(−1.1, −0.6)	< 0.05*
LA GLS (%)	−14.7(−20.4,−13.6)	−16.9(−20.6,−10)	1.00
LA GRS (%)	24.1(23.2,43.5)	32.7(17.4,44.9)	0.75
Total LA EF (%)	48(40.6,49.7)	49(42.5,51)	0.62
Passive LA EF (%)	24.5(16.1,33.3)	31.4(26.3,32.6)	0.44
Active LA EF (%)	28.5(22.6,33.6)	25(17.6,28.6)	0.34
LVEF (%)	58.5(55,68.7)	62(55,71)	0.75
LV GLS (%)	−8.9(−16.4, −7.8)	−14.7(−16.4, −4.7)	0.62
LV GCS (%)	−19.5 (−20.9, −16.4)	−16.4 (−18.4,−16.4)	0.25
LV GRS (%)	−19.5 (−20.9, −16.4)	−16.5(−18.4,−12.4)	0.25
Native T1 mapping	1073 (1047,1095)	1068 (920,1115)	0.82

*LACI* left atrioventricular coupling index, *LAV max* left Atrial Volume Maximum, *LAV min* left Atrial Volume Minimum, *LA ξs* Left atrial longitudinal reservoir strain, *LA ξe* Left atrial passive strain, *LA ξa* Left atrial active strain, *LA SRs* Left atrial longitudinal peak positive strain rate, *LA SRe* Left atrial peak early negative strain rate, *LA SRa* Left atrial peak late negative strain rate, *LA* left atrium, *GLS* Global longitudinal strain, *GCS* Global circumferential strain, *GRS* global radial strain. *EF* ejection fraction, *LV* left ventricle. Significant values are in bold.

In addition to LACI and LA ξa, we performed ROC curve analyses for other variables, including LAVmin, LVEF, LA ξs, and LV GLS to assess their predictive power for arrhythmia occurrence. LAVmin yielded an AUC of 0.71, LVEF had an AUC of 0.66, and LA ξs had an AUC of 0.74. These results are presented in (Table 7) and suggest that LACI and LA ξa may offer superior discriminatory value in identifying patients at higher clinical risk.

**Table 7:** Comparison of the discriminatory ability of different cardiac parameters for arrhythmia prediction in MVP patients.

Parameters	AUC	Sensitivity	Specificity
LACI (%)	0.84	100	60
LA ξa (%)	0.89	100	60
LAVmin indexed (ml/m^2^)	0.71	75	50
LVEF (%)	0.66	50	60
LA ξs (%)	0.74	75	55
LV GLS (%)	0.70	70	50

*LACI * left atrioventricular active strain, *LA ξa *Left atrial longitudinal reservoir strain, * LAVmin indexed *minimum left atrial volume indexed to body surface area, *LVEF *left ventricular ejection fraction, *LA ξs *Left atrial longitudinal reservoir strain, *LV GLS *left ventricle global longitudinal strain.

### T1 mapping correlation

Native T1 values were significantly higher in MVP patients compared to controls [934(924–958) ms] vs. [1062(920–1100) ms], *p* < 0.05. In our cohort, native T1 mapping demonstrated significant correlations with several markers of cardiac function. Specifically, native T1 values showed a moderate positive correlation with LACI (*r* = 0.53, *p* < 0.05) and LV GLS (*r* = 0.52, *p* < 0.05), suggesting an association between diffuse myocardial fibrosis and left atrioventricular coupling. A significant inverse correlation was also observed with LV GRS (*r* = −0.46, *p* < 0.05), reflecting reduced radial contractility. Additionally, native T1 values were positively correlated with left atrial booster strain (ξa) (*r* = 0.45, *p* < 0.05) and negatively correlated with booster strain rate (SRa) (*r* = −0.51, *p* < 0.05), indicating subtle alterations in active atrial contraction. Other volumetric and strain parameters, including LVEF and LVEDVI, showed no significant correlation with native T1 mapping. These results are presented in (Table 8).

**Table 8:** Native T1 Mapping Correlation with left ventricular and left atrial volumetric and strain indices.

Parameters	*R* value	*P* value
Native T1 mapping		
LACI	0.53	< 0.05*
LVEF	0.06	0.79
LVEDVI	-0.04	0.88
LV GLS (%)	0.52	< 0.05*
LV GCS (%)	0.26	0.26
LV GRS (%)	-0.46	< 0.05*
LAV max indexed	0.03	0.99
LAV min indexed	0.04	0.88
Total LA EF	0.07	0.76
Passive LA EF	0.15	0.53
Active LA EF	0.08	0.72
LA GLS (%)	-0.36	0.12
LA GRS (%)	0.29	0.21
LA ξs (reservoir) (%)	0.27	0.25
LA ξe (conduit) (%)	-0.03	0.89
LA ξa (booster) (%)	0.45	< 0.05*
LA SRs (reservoir) (s-1)	0.37	0.11
LA SRe (conduit) (s-1)	-0.37	0.11
LA SRa (booster) (s-1)	-0.51	< 0.05*

* Statistically significant, *LACI* left atrioventricular coupling index, *LV* left ventricle*, EF *ejection fraction, *EDVI* end-diastolic volume indexed, *GLS* left ventricle global longitudinal strain, *GCS* left ventricle global circumferential strain, *GRS*left ventricle global radial strain, *LAV max *left Atrial Volume Maximum, *LAV min* left Atrial Volume Minimum, *LA*left atrium, *LA ξs* Left atrial longitudinal reservoir strain, *LA ξe* Left atrial passive strain, *LA ξa* Left atrial active strain, *LA SRs* Left atrial longitudinal peak positive strain rate, *LA SRe* Left atrial peak early negative strain rate, *LA SRa* Left atrial peak late negative strain rate.

## Discussion

This study investigated left atrioventricular (LA-LV) coupling in pediatric patients with isolated mitral valve prolapse (MVP) and moderate mitral regurgitation, utilizing CMR-derived volumetric and strain metrics as well as the left atrioventricular coupling index (LACI). We also relied on the native T1 mapping technique to provide an insight into possible myocardial fibrosis. Our results indicate that functional impairments in both the left atrium and ventricle occur early, even with a preserved ejection fraction, thereby supporting the idea that these patients show myocardial abnormalities relative to healthy counterparts.

When compared to healthy controls, MVP patients exhibited greater ventricular end-diastolic volume index, left ventricular wall mass, and minimum left atrial volume. Notably, LA reservoir strain was significantly lower in MVP patients, in line with previous research by Tastet et al., which showed that MVP patients with LA enlargement had diminished reservoir function and increased LVEDVI. This suggests that LA dysfunction may stem from intrinsic myocardial abnormalities rather than solely from MR volume overload^8^.

To unveil the mechanics of LA-LV coupling, LACI was assessed in all patients and controls, and strain parameters for both the left atrium and ventricle. Studying coupling was presented in our work using the volumetric analysis of the left atrium and the left ventricle, as demonstrated in LACI. Second, it was studied using the correlations between the left atrial and the left ventricular strain parameters, aiming to emphasize the subclinical impairment if present. LACI did not differ significantly between patients and controls, giving the idea that, alongside a preserved EF, those patients seem to have a good LA-LV synchrony. On the other hand, using more advanced parameters, including T1 Native mapping and strain analysis, provided a more sophisticated and deeper insight that a subclinical LA-LV dysynchrony may be present. There was a significant difference between patients and control subjects regarding the T1 Native parameters, denoting a subclinical left ventricular myocardial affection. Moreover, strain analysis of all patients and controls varied significantly, suggesting a superadded atrial myocardial deformation.

While LACI did not differ significantly between MVP patients and controls, it was elevated in patients who experienced clinical events, suggesting potential prognostic relevance. Strain analysis uncovered subclinical myocardial alterations that were associated with clinical events. The predictive values (100% sensitivity) for LACI and LA ξa, while promising, are derived from a very small event group and are likely overestimates, reinforcing the need for validation. These results highlight the value of advanced functional imaging in detecting early cardiac involvement in pediatric MVP and suggest that strain-based assessment may enhance risk stratification in this population.

While we performed comparative analyses of multiple LA and LV strain parameters between patients with and without clinical events, only LACI and LA active strain (ξa) demonstrated significant differences. Other strain parameters, including LA reservoir strain (ξs), conduit strain (ξe), and LV strain indices (GRS, GCS), did not reach statistical significance in relation to clinical events. This may be due to the limited number of clinical events in our cohort (*n* = 4), which restricts statistical power and increases the risk of Type II error. Alternatively, it is possible that in early-stage or moderate MVP, mechanical dysfunction is more localized to specific phases of atrial activity (e.g., contraction) and not yet reflected in broader strain abnormalities. However, it is important to note that these findings are derived from a limited sample size, with only four clinical events recorded. Therefore, these results should be regarded as preliminary. To validate the prognostic significance of LACI in this patient population, further research involving larger, prospective cohorts is necessary.

The strong positive correlations between LACI and both the reservoir strain of the left atrium and LV GCS in our study are understandable, as these strain measurements provide insights into the compliance of the left atrium and ventricle, which are typically affected in these patient populations. In conclusion, both the left atrium and ventricle show similar impairments in patients with isolated mitral valve prolapse.

Such synchrony and mechanics were supported by studying the phasic relationship between the LA function and LV function, which seemed to be synchronized. Left atrial enlargement and impairment were found to be concurrent with left ventricular dysfunction. LA reservoir function was shown to decrease as LV end-diastolic volume increased. On the other hand, it increased as the LV ejection fraction increased. As for LA contractile function, it increased as the LV ejection fraction increased. A study conducted by Rajes et al. on patients with mitral valve prolapse as a cause of primary mitral regurgitation revealed that, through strain rate analysis, patients had higher strain rates than controls^15^. Also, MVP patients with no or mild mitral regurgitation in a study by Taset et al. demonstrated lower reservoir strain rates^8^. These studies imply that intrinsic myocardial alterations may contribute to the atrial and ventricular abnormalities seen in MVP regardless of the mitral valve regurgitation degree.

Upon conducting linear logistic analysis to determine which LV parameters were independently affected by LA strain parameters, the LA-LV coupling, and harmonic affection revealed that LA reservoir strain (ξs) was independently associated with LVEF and LV GRS, highlighting the functional interdependence between left atrial filling capacity and left ventricular contractile performance. The LA conduit strain (ξe) was found to be influenced independently by LVEF and LV GRS. This relationship provides insight into the hemodynamic interplay between the LA and LV during early diastole, thus emphasizing that LA conduit function is not solely a reflection of atrial properties but is significantly influenced by ventricular compliance and function. Additionally, the booster phase (ξa) appeared to be independently affected by LVEF, indicating that subtle impairments in ventricular function can directly influence atrial contractility. These patterns emphasize the interdependence of atrial and ventricular function and suggest that strain-based parameters may serve as early markers of remodeling.

However, the absence of MVP patients with reduced LVEF limits the ability to assess how strain metrics behave across the full spectrum of ventricular function. Several studies have shown that strain parameters of both LA and LV are strongly associated with reduced LVEF or with disease severity, even when EF is preserved. For example, Pellicori et al. conducted a large study of heart failure patients stating that LA reservoir strain worsens in those with more advanced diastolic dysfunction regardless of EF status^16^. Similarly, Rossi et al. assessed atrioventricular coupling by CMR in mixed cardiomyopathy populations, demonstrated that in subjects with reduced LVEF, LV strain impairment is more profound, and LA strain adds incremental information beyond traditional metrics^17^. Another study in functional mitral regurgitation by Kusunose et al. revealed that reduced peak LA longitudinal strain had prognostic value in those with LVEF < 50%^18^. Even in pediatric populations, such as in hypertrophic cardiomyopathy, a study by Jefferies et concluded that LA reservoir and conduit strain are reduced before typical volumetric, or EF changes are prominent^19^.

The left atrioventricular coupling index (LACI) has recently gained recognition as a marker of mechanical coordination between the left atrium and ventricle. A significant adult study by Essayagh et al. demonstrated that elevated LACI independently predicted mortality in MVP patients, even when adjusting for MR severity and other factors^9^. While most current data on LACI is from adult populations, our research is among the first to investigate its implications in pediatric MVP.

To deepen our understanding of LACI, we compared our findings with adult MVP literature and explored its behavior in relation to MR severity and clinical outcomes. Previous research on adult MVP populations has demonstrated that altered LACI correlates with adverse clinical outcomes, including arrhythmias and myocardial remodeling. For instance, adult studies as Tastet et al. reported significantly elevated LACI values associated with impaired left atrial and ventricular function, suggesting progressive atrioventricular uncoupling^8^.

Although we observed impaired left atrioventricular coupling reflected by correlations between LACI and LA and LV function and subtle impairment in left atrial strain parameters, the absolute LACI values did not significantly differ from healthy controls. However, the presence of a notably higher LACI in patients who experienced clinical events suggests potential prognostic value in younger populations as well and warrants further longitudinal assessment.

This means that while LACI may appear preserved in the broader MVP cohort, it may still reflect subclinical dysfunction or compensatory atrial remodeling in at-risk individuals. One possible explanation is that this difference may reflect compensatory mechanisms unique to the pediatric myocardium, maintaining LACI within the normal range or earlier disease stages prior to extensive remodeling seen in adults, manifesting as arrhythmias or further dysfunction. Moreover, developmental factors such as myocardial compliance and growth may influence the interpretation of LACI differently across age groups, which is consistent with prior pediatric CMR studies, as Figliozzi et al.^7^. Our findings thus underscore the need for age-specific normative data and caution when extrapolating adult MVP insights to pediatric populations. Further longitudinal studies are warranted to clarify how LACI evolves from childhood through adulthood in MVP and its potential prognostic value.

Additionally, the lack of group-level significance may be partly due to sample size limitations. Therefore, LACI should not be interpreted in isolation, and combining it with LA strain parameters may provide a more nuanced assessment of atrioventricular coupling and its clinical implications.

Although the arrhythmias recorded in our study were benign and did not require pharmacologic intervention, their occurrence in MVP patients highlights the need for close surveillance, especially in those with elevated LACI or impaired LA strain parameters. The conservative management approach reflects current pediatric arrhythmia guidelines for benign ectopy in structurally normal hearts.

Previous studies have shown that myocardial fibrosis is frequently observed in individuals with MVP, and it has been linked to instances of sudden cardiac death. Morningstar et al. demonstrated that LV biopsies, which were performed on surgical patients with mitral valve prolapse from the peripapillary regions, infero-basal LV wall, and apex, revealed regionalized fibrosis in the peripapillary myocardium^4^.

Myocardial fibrosis in MVP patients has been previously investigated in patients with moderate to severe MR using the late gadolinium enhancement MRI techniques. However, the strain analysis of such myocardia has not been investigated specifically in the pediatric population. Kitkungvan and colleagues conducted a study on 356 patients with MR, with 56% of patients having moderate or greater MR. They found that patients with mitral valve prolapse had a higher occurrence of myocardial fibrosis detected by late gadolinium enhancement compared to other forms of MR (37% vs. 7%; *p* < 0.001)^20^.

Measuring extracellular volume (ECV) as an indicator for subclinical fibrosis has also been addressed, where elevated values have also been linked to the onset of symptoms and clinical deterioration. In comparison to late gadolinium enhancement, an increased extracellular volume exhibited a similar pattern in patients with MVP and those without, but with MR. This suggests that late gadolinium enhancement may more accurately represent the pathophysiological changes associated with MVP, while an elevated ECV better indicates volume overload regardless of the underlying cause^21,22^.

In our study, only native T1 mapping was assessed as it was the only non-contrast tissue characterization technique consistently available across the included cases. Given that contrast administration is not routinely performed for the evaluation of mitral valve prolapse in our institution, particularly in pediatric patients, late gadolinium enhancement (LGE), post-contrast T1 mapping, and ECV measurements were not feasible. Furthermore, the study’s retrospective design limited our ability to standardize or acquire additional contrast-enhanced imaging. Therefore, native T1 mapping was used as a surrogate marker for diffuse myocardial fibrosis, providing valuable insights into myocardial tissue characteristics without the need for gadolinium-based contrast agents. Elevated native T1 values observed in MVP patients suggest the presence of diffuse myocardial fibrosis.

These findings are consistent with prior research where native T1 mapping was used to detect myocardial fibrosis in various cardiac conditions. For example, Schelbert et al. demonstrated significantly elevated native T1 values in patients with hypertrophic cardiomyopathy compared to healthy controls, indicating diffuse fibrosis^23^. Similarly, Puntmann et al. reported increased native T1 times in dilated cardiomyopathy, correlating with adverse clinical outcomes^10^.

The significant associations between native T1 values and both atrial and ventricular strain parameters suggest that diffuse myocardial fibrosis may underlie early functional impairments in pediatric MVP patients. Elevated T1 values, which serve as a non-contrast surrogate for interstitial fibrosis, correlated with reduced LV strain (particularly GRS) and altered LA contractile function (ξa and SRa), supporting the hypothesis that myocardial tissue changes precede overt volumetric dysfunction. The observed correlation with LACI further emphasizes the interplay between myocardial structure and atrioventricular coupling, reinforcing its potential prognostic role. This aligns with the known arrhythmogenic role of myocardial fibrosis in MVP and further underscores the potential utility of combining tissue characterization with functional strain analysis for early risk detection in pediatric populations. However, the absence of correlation with traditional volumetric indices and preserved LVEF underscores the importance of advanced imaging in detecting subclinical myocardial involvement.

In MVP specifically, Dabir et al. found elevated native T1 values indicating myocardial tissue alterations, highlighting the potential for early myocardial involvement in MVP patients^11^.

While native T1 mapping offers valuable insights, advanced tissue characterization techniques such as post-contrast T1 mapping and extracellular volume (ECV) quantification could provide more specific assessments of myocardial fibrosis. Future studies at larger cohorts incorporating these modalities may further clarify the pathophysiology of MVP-related myocardial remodeling and its clinical implications.

This study has several limitations. First, the relatively small sample size may restrict the generalizability of the results and increase the risk of Type II error, particularly when evaluating subgroup differences or rare clinical events. Second, the study was conducted at a single tertiary care center, which may limit the external validity of the findings. Although our findings offer initial insights into atrioventricular coupling and strain patterns in children with moderate MR and MVP, further multicenter studies are needed to validate these results and evaluate their relevance in larger populations. Third, the retrospective design may be subject to selection bias and limitations in data completeness. Additionally, the number of clinical events (*n* = 4) was too low to allow for robust predictive modeling or risk stratification; the presence of arrhythmias in a small subset of patients may affect myocardial strain and coupling indices. Therefore, stratified analyses were not feasible. Furthermore, although LACI and LA ξa demonstrated high predictive performance, including 100% sensitivity in our analysis, these findings are based on a very small number of clinical events and are likely overestimates; therefore, validation in larger and more diverse patient populations is essential. Future studies with larger cohorts and detailed characterization of arrhythmias are necessary to clarify these relationships further. Also, the follow-up period was relatively short, and longer-term outcomes, especially arrhythmic progression or changes in strain parameters, remain unknown. Studying pediatric patients with isolated mitral valve prolapse with only a moderate degree of mitral regurgitation; Consequently, the findings may not be generalizable to adult populations, patients with severe mitral regurgitation, or those with additional valvular pathologies. Furthermore, this study exclusively included pediatric MVP patients with preserved left ventricular ejection fraction, limiting insights into myocardial dysfunction in those with reduced LVEF. Consequently, the effects of clinical treatments and surgical interventions on this subgroup could not be evaluated. Future research should focus on patients with reduced LVEF to better understand treatment impacts and provide more clinically actionable guidance. Also, Future research involving larger and more diverse cohorts is warranted to assess the applicability of left atrioventricular coupling indices across different patient groups.

## Conclusions

This study provides preliminary evidence that pediatric patients with isolated mitral valve prolapse and moderate mitral regurgitation demonstrate early signs of left atrial and ventricular dysfunction despite preserved ejection fraction. Strain imaging revealed subclinical impairments in LA reservoir, conduit, and booster functions, which were closely linked to LV performance. The observed associations between atrial strain components and left ventricular structural and functional parameters suggest potential interdependence in remodeling. Native T1 mapping further indicated diffuse myocardial changes, supporting the presence of early fibrosis. These findings highlight the importance of incorporating strain imaging and tissue characterization in the assessment of pediatric MVP, and suggest that LACI, in combination with strain parameters, may aid in risk stratification.

## Data Availability

All the scientific data are available and presented in the manuscript. The source data are available upon reasonable request to the corresponding author.

## Abbreviations

MVP: Mitral valve prolapse
MR: Mitral regurgitation
LV: Left ventricle
LVEF: Left ventricular ejection fraction
LV-EDVI: Left ventricular end-diastolic volume indexed to BSA
LV EDWM: Left ventricular end-diastolic wall mass
LV EDWMI: Left ventricular end-diastolic wall mass indexed to body surface area
LVESVI: Left ventricular end-systolic volume indexed to body surface area
LVESV: Left ventricular end-systolic volume
LA: Left atrium
LACI: Left atrioventricular coupling index
CMR: Cardiovascular magnetic resonance (cardiac magnetic resonance)
ECHO: Echocardiography
ECG: Electrocardiogram
SSFP: Steady-state free precession
TR: Time of repetition
TE: Time to echo
FOV: Field of view
GLS: Global longitudinal strain
GRS: Global radial strain
GCS: Global circumferential strain
LAV max: Left atrial volume at maximum (end of LV systole)
LAV min: Left atrial volume at minimum (end of LV diastole)
LAV pac: Left atrial volume pre-atrial contraction
LAEF: Left atrial ejection fraction
CMR-FT: Cardiac magnetic resonance imaging- feature tracking
ξs: Longitudinal reservoir strain
ξe: Passive (conduit) strain
ξa: Active (booster) strain
SRs: Reservoir strain rate
SRe: Conduit strain rate
SRa: Booster strain rate
MOLLI: Modified look-locker inversion recovery
ECV: Extracellular volume
ICD: Implantable cardioverter defibrillator
IRB: Institutional review board
ROC: Receiver operating characteristic
AUC: Area under the curve
CI: Confidence interval
